# Preclinical investigation of ibrutinib, a Bruton's kinase tyrosine (Btk) inhibitor, in suppressing glioma tumorigenesis and stem cell phenotypes

**DOI:** 10.18632/oncotarget.11572

**Published:** 2016-08-24

**Authors:** Li Wei, Yu-Kai Su, Chien-Min Lin, Tsu-Yi Chao, Shang-Pen Huang, Thanh-Tuan Huynh, Hsun-Jin Jan, Jacqueline Whang-Peng, Jeng-Fong Chiou, Alexander T.H. Wu, Michael Hsiao

**Affiliations:** ^1^ The Ph.D. Program for Translational Medicine, College of Medical Science and Technology, Taipei Medical University and Academia Sinica, Taipei, Taiwan; ^2^ Division of Neurosurgery, Department of Surgery, Wan Fang Hospital, Taipei Medical University, Taipei, Taiwan; ^3^ Department of Neurology, School of Medicine, College of Medicine, Taipei Medical University, Taipei, Taiwan; ^4^ Division of Neurosurgery, Department of Surgery, Shuang Ho Hospital, Taipei Medical University, Taipei, Taiwan; ^5^ Graduate Institute of Clinical Medicine, College of Medicine, Taipei Medical University, Taipei, Taiwan; ^6^ Department of Neurology, Taiwan Adventist Hospital, Taipei, Taiwan; ^7^ Center for Molecular Biomedicine, University of Medicine and Pharmacy, HoChiMinh City, Vietnam; ^8^ The Department of Medical Laboratory and Biotechnology, Central Taiwan University of Science and Technology, Taichung, Taiwan; ^9^ Division of Cancer Center, Wan Fang Hospital, Taipei Medical University, Taipei, Taiwan; Center of Excellence for Cancer Research, Taipei Medical University, Taipei, Taiwan; ^10^ Department of Radiation Oncology, Taipei Medical University Hospital, Taipei, Taiwan; ^11^ Department of Radiology, School of Medicine, College of Medicine, Taipei Medical University, Taipei, Taiwan; ^12^ Genomics Research Center, Academia Sinica, Taipei, Taiwan; ^13^ Department of Biochemistry, College of Medicine, Kaohsiung Medical University, Kaohsiung, Taiwan

**Keywords:** glioma, cancer stem cells, Bruton's tyrosine kinase, ibrutinib

## Abstract

Standard interventions for glioma include surgery, radiation and chemotherapies but the prognosis for malignant cases such as glioblastoma multiforme remain grim. Even with targeted therapeutic agent, bevacitumab, malignant glioma often develops resistance and recurrence. Thus, developing alternative interventions (therapeutic targets, biomarkers) is urgently required. Bruton's tyrosine kinase (Btk) has been long implicated in B cell malignancies but surprisingly it has recently been shown to also play a tumorigenic role in solid tumors such as ovarian and prostate cancer. Bioinformatics data indicates that Btk is significantly higher in clinical glioma samples as compared to normal brain cells and Btk expression level is associated with stage progression. This prompts us to investigate the potential role of Btk as a therapeutic target for glioma. Here, we demonstrate Btk expression is associated with GBM tumorigenesis. Down-regulation of Btk in GBM cell lines showed a significantly reduced abilities in colony formation, migration and GBM sphere-forming potential. Mechanistically, Btk-silenced cells showed a concomitant reduction in the expression of CD133 and Akt/mTOR signaling. In parallel, Ibrutinib (a Btk inhibitor) treatment led to a similar anti-tumorigenic response. Using xenograft mouse model, tumorigenesis was significantly reduced in Btk-silenced or ibrutinib-treated mice as compared to control counterparts. Finally, our glioma tissue microarray analysis indicated a higher Btk staining in the malignant tumors than less malignant and normal brain tissues. Collectively, Btk may represent a novel therapeutic target for glioma and ibrunitib may be used as an adjuvant treatment for malignant GBM.

## INTRODUCTION

Malignant brain tumors are challenging to manage clinically due to the nature of highly resistant to chemotherapy and radiotherapy [1]. Recurrence following standard therapeutic regimens, mainly including surgery, radiation therapy, and adjuvant chemotherapy is virtually inevitable [2]. The standard chemotherapeutic agent such as temozolomide (TMZ) promotes DNA damage and disrupts the brain tumor cell mitotic machinery. However, it has only limited effect in prolonging patient survival [3]. Accumulating evidence has suggested that malignant glioma stem cells (GSCs), intrinsic and/or therapy-induced, drive tumor formation and treatment resistance [4]. Thus, identifying agents which can target and eliminate GSCs may improve current therapeutic efficacy in GBM patients.

The Bruton's tyrosine kinase (Btk) family kinases consists of non-receptor tyrosine kinases, including Btk/Atk, Bmx/Etk, Itk/Emt/Tsk, and Tec. Accumulating evidence has suggested that Btk family kinases, like Src-family kinases, may play central and diverse modulatory roles in many important cellular processes [5, 6]. Btk regulates key signaling pathways such as PI3K, PLCγ and PKC which suggest their involvements in cellular growth, differentiation and apoptosis. Recently, a new role of this family of kinases is emerging in regulating cytoskeletal reorganization and cell motility [5, 7]. Previously, Btk dysregulation has been mainly attributed to the development of B-cell malignancy. However, recent studies have shown its involvement in solid tumors such as breast, ovary, prostate and lung [8–10]. Together, these findings provide novel link between Btk and cellular processes such as cellular growth, differentiation, motility and inflammation, all of which if dysregulated, contribute to the progression of cancer.

Interestingly, Btk is recently linked to the activation of NLRP3 inflammasome in ischaemic stroke [11] and as a potential therapeutic target. This finding suggests that Btk is important in neurological development and diseases. However, the role of Btk in the GSC generation has not been explored. This prompts us to investigate Btk's role in GSC generation and associated malignant phenotypes and mitigating strategies based on Btk inhibition. Here, we first utilized bioinformatics tool and observed a significantly elevated Btk expression in glioma patients as compared to normal brain tissue. Increased Btk expression in GBM spheres was associated with increased stemness, metastatic potential and temozolomide resistance. Btk-silenced GBM cells exhibited a significantly lower ability to form colonies, GBM spheres and metastasize. Notably, ibrutinib (a Btk inhibitor) treatment also led to a similar suppressive effect in GBM malignant phenotypes as observed under gene-silencing conditions. Furthermore, GBM xenograft models demonstrated that down-regulation of Btk via gene-silencing or ibrutinib treatment resulted in a significant reduction in GBM tumorigenesis. Finally, brain tumor tissue microarray data indicated that Btk expression was associated with the disease stage. Collectively, we have provided evidence for targeting Btk using ibrutinib as an alternative and/or adjuvant intervention to the existing therapeutic regimen.

## RESULTS

### Elevated Btk expression in glioma cells

**Figure 1:**
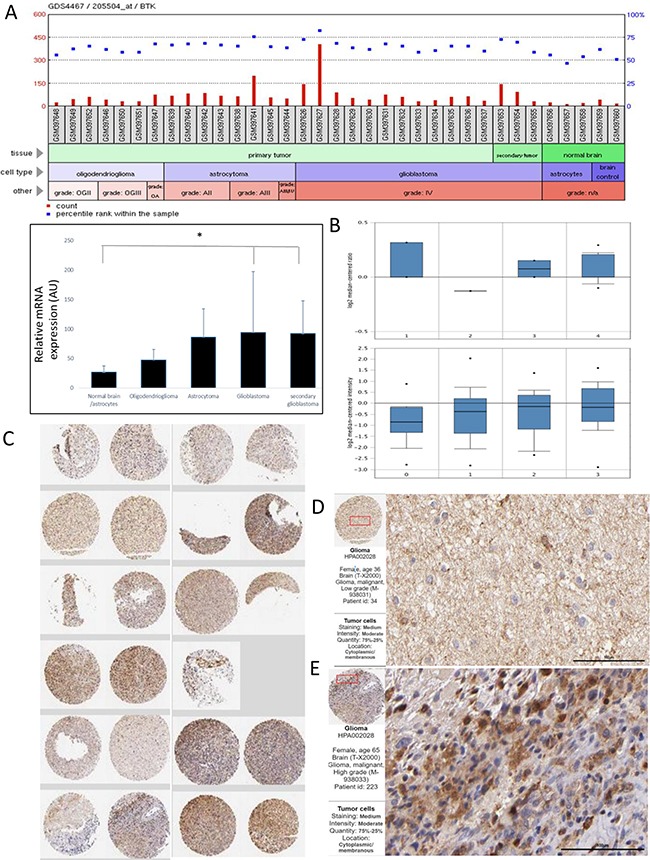
An elevated Btk expression is found in clinical GBM samples **A.** Btk expression is elevated in advanced GBM clinical samples. (A) A GEO expression database used in this analysis contained different sample groups from primary tumor (subdivided into 3 categories, oligodendrioglioma, astrocytoma), secondary GBM and normal brain/astrocytes. Statistical analysis of Btk expression between tumor versus normal/astrocytes groups revealed that GBM and secondary GBM samples contain a significantly higher level of Btk mRNA as compared to normal brain and astrocyte samples (Student T-test, *P<0.05). GEO dataset used GDS4467. **B.** Yamanaka Brain dataset (GSE4381) analysis demonstrates the elevated Btk expression in different brain tumor samples. 1. Anaplastic Astrocytoma (N=4); 2. Anaplastic Oligoastrocytoma (N=1); 3. Anaplastic Oligodendroglioma (N=2); 4. Glioblastoma (N=22). In the 22 GBM patients, the higher the Btk level appears to be associated with death. The lower panel represents another dataset (Sun brain dataset, GSE4290) analysis also suggests an association between Btk mRNA level and GBM progression. 0. No grade (N=23); 1. Grade 2 (N=45); 2. Grade 3 (N=31); 3. Grade 4 (N=81). **C.** Btk protein expression was also found elevated in clinical tissue microarray, as indicated by proteinatlas database. 2 out of 9 samples showed high intensity while 9 out of 12 samples exhibited moderate staining. The staining of Btk was mainly identified in the cytoplasm and membrane. Representative and magnified micrograph of a low grade **D.** and high grade **E.** GBM patient sample respectively, demonstrating elevated Btk expression. Antibody used HPA002028; http://www.proteinatlas.org/ENSG00000010671-BTK/cancer/tissue/glioma.

Our preliminary search using public databases indicated Btk expression is elevated in advanced GBM clinical samples (Figure 1A). Notably, a GEO expression database (GDS4467) [12] used in this analysis contained different sample groups including primary tumor (subdivided into 3 categories, oligodendrioglioma, astrocytoma), secondary GBM and normal brain/astrocytes. Using this database, our statistical analysis showed that GBM and secondary GBM samples contained a significantly higher level of Btk mRNA as compared to normal brain and astrocyte samples (Figure 1A). In addition, Yamanaka Brain dataset (GSE4381) [13] consists of 22 glioblastoma, 4 anaplastic astrocytoma, 2 anaplastic oligodendroglioma, and 1 anaplastic oligoastrocytoma samples were analyzed using Agilent Human 1 cDNA microarrays. Based on the selected threshold (p value = 1E-4) and fold change at least at 2 and top 1% gene rank, Btk was found significantly elevated in the anaplastic astrocytoma, anaplastic oligodendroglioma and glioblastoma (upper panel, Figure 1B). When the GBM samples are grouped by one year survival, samples from the deceased express higher level of Btk. In addition, we used another larger database, Sun Brain database (GSE4290) consisting of 157 brain and CNS tumors and 23 normal brain samples, which was analyzed on Affymetrix U133 Plus 2.0 microarrays. A positive association between Btk mRNA level and tumor grade was also identified (lower panel, Figure 1B). ProteinAtlas database provided additional evidence that a higher Btk protein expression was detected in clinical tissue microarray. For instance, 2 out of 9 samples showed high intensity while 9 out of 12 samples exhibited moderate staining. The staining of Btk was mainly identified in the cytoplasm and membrane. Representative and magnified micrograph of a low grade (C) and high grade (D) GBM patient sample demonstrating elevated Btk protein expression.

### Btk expression is associated with increased malignant phenotypes and stemness

**Figure 2:**
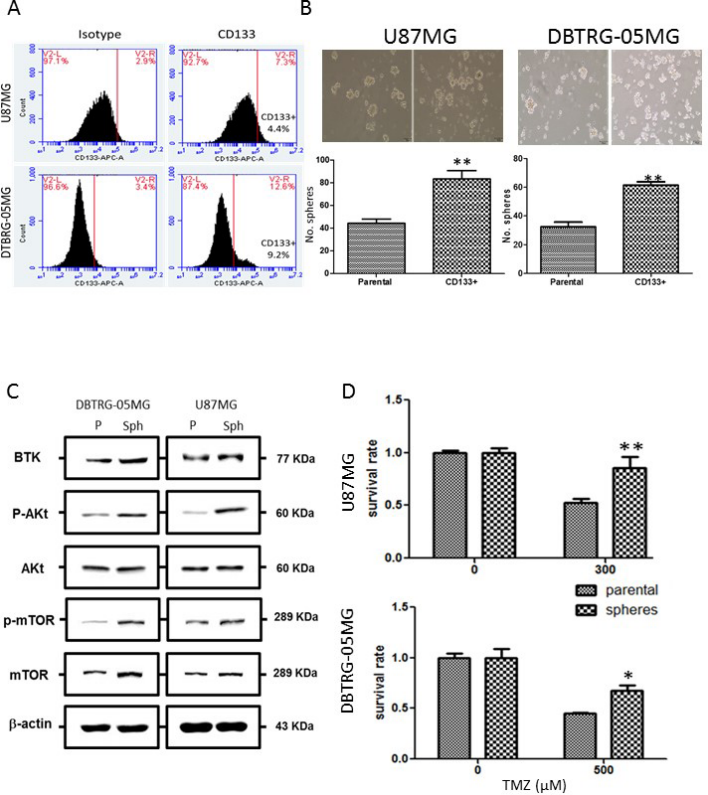
Increased Btk, stemness marker expression and temozolomide resistance in GBM spheres **A.** Identification of CD133+ population of GBM cells using flow cytometric analysis. CD133+ cell population was identified in both U87MG and DTBRG-05MG cell lines (approximately 4.4% and 9.2% respectively). **B.** CD133+ GBM cells when cultured under serum-deprived conditions showed an increased propensity for GBM sphere formation as compared to their CD133- parental counterparts. **C.** Comparative Western blots analysis between GBM parental cells and GBM spheres. In both cell lines, Btk and oncogenic Akt/mTOR signaling expression were both found higher in the GBM spheres than their parental counterparts. **D.** GBM GBM spheres exhibited enhanced temozolomide resistance. The IC50 value was found to be approximately 300 and 500 μM for U87MG and DBTRG-05 cells respectively. U87MG and DBTRG-05 GBM spheres remained approximately 85.7% and 67.8% viable at the IC_50_ concentrations of their respective parental counterparts.

Btk's role in GSC generation/maintenance was examined using U87MG and DBTRG-05MG human GBM cell lines. First, the percentage of CD133+ GBM cells were determined and subsequently isolated using FACS technique (Figure 2A). CD133+ U87MG (approximately 4.4%) and DBTRG-05MG (approximately 9.2%) cells were isolated and cultured under serum-deprived condition. These cells formed GBM spheres more efficiently as compared to their CD133- parental counterparts (Figure 2B). Comparative Western blots of total cell lysates obtained from parental and spheres indicated a higher Btk expression in the GBM spheres generated from both cell lines (Figure 2C); phosphorylated form of Akt and mTOR (p-Akt and p-mTOR respectively) were also up-regulated in the spheres (Figure 2C). We also observed that GBM spheres exhibited a significantly higher resistance against temozolomide treatment as approximately 85.7% of U87MG spheres remained viable at 300 μM (IC_50_ for parental counterparts) and 67.8% for DBTRG-05MG spheres under 500 μM temozolomide treatment (Figure 2D). These observations suggest a link between Btk expression and the generation of GBM spheres and associated drug resistance.

### Silencing Btk was associated with decreased GBM tumorigenesis and stemensess

**Figure 3:**
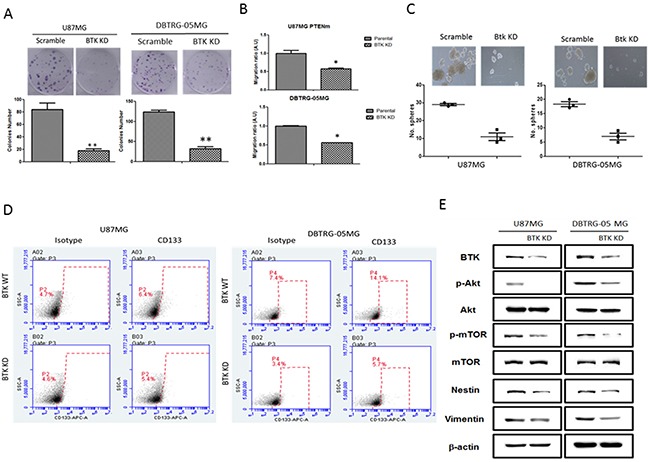
Btk-silencing resulted in decreased malignant GBM phenotypes and stemness **A.** Btk knocked down U87MG and DBTRG-05MG cells exhibited a significantly decreased colony-forming ability. **B.** Btk-silenced U87MG and DBTRG-05MG cells demonstrated a decreased migratory ability as compared to their counterparts. **C.** Down-regulation of Btk resulted in a significantly reduced sphere-forming ability in both cell lines. **D.** Flow cytometric analysis showed that Btk-silencing was associated with reduced the percentage of CD133^+^ U87MG and DBTRG-05MG (approximately 4.4% and 0.9% reduction respectively). **E.** Comparative Western blots between parental and Btk-knocked down cells. Btk-downregulation was associated with reduced expression level of stemness genes including c-Myc, Nestin, as well as the oncogenic Akt/mTOR signaling and the EMT marker, Vimentin.

To examine the association between Btk expression, GBM tumorigenesis and GSC generation, Btk expression was down-regulated by gene-silencing technique in both U87MG and DBTRG-05MG cells. Btk-silenced U87MG and DBTRG-05MG cells exhibited a significantly reduced tumorigenic phenotypes. First, the colony forming ability was significantly reduced in both Btk-silenced U87MG and DBTRG-05MG cells, approximately 4-fold decrease in both cell lines (Figure 3A). Second, the migratory ability was significantly suppressed upon Btk-silencing (Figure 3B). Notably, Btk-silenced U87MG and DBTRG-05MG cells showed a markedly reduced number of GBM spheres generated (Figure 3C). When we examined the association between Btk and CD133 (stemness marker) expression using flow cytometry. Btk-silenced U87MG and DBTRG-05MG showed a significantly lower percentage of CD133+ cell population. For instance, CD133+ cell percentage decreased from 1.7% to 0.8% in U87MG cells and 6.7% to 2.3% in DBTRG-05MG (Figure 3D). Western blot analysis provided support to the observed phenomena where reduced Btk expression was associated with a decreased level of Btk, Nestin (both stemness and EMT marker), CD133 (stemness marker) and Akt/mTOR signaling pathway as well as the EMT marker vimentin (Figure 3E).

### Btk inhibitor ibrutinib suppressed tumorigenesis via Btk downregulation

**Figure 4:**
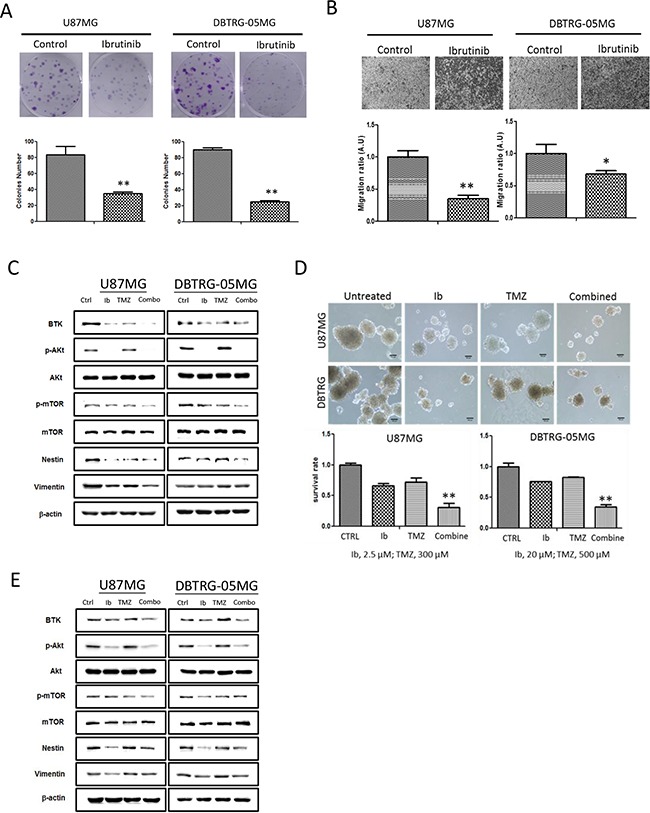
Ibrutinib suppressed GBM tumorigenic ability resembling Btk-gene silenced GBM cells **A.** The colony forming ability of both U87MG and DTBRG-05MG cells were significantly suppressed at low concentration of Ib (0.5 uM at IC10 for U87MG while 4 uM at IC_20_ for DTBRG-05MG cells). **B.** Ibrutinib treatment significantly decreased the migratory ability of both GBM cell lines (at 0.25uM and 4uM for U87MG and DTBRG-05MG respectively, these concentrations represent the IC_20_ values). **C.** Western blots of total protein lysates collected from both U87MG and DTBRG-05MG cells treated with ibrutinib (Ib), temozolomide (TMZ), and two drugs combined (Combo). The treatment conditions were as the follows: U87MG (Ib,10μM; TMZ300 μM); DBTRG-05MG (Ib, 30μM; TMZ 500 μM). The expression level of Btk, p-Akt and p-mTOR was significantly reduced by Ib treatment while TMZ only treatment moderately reduced Btk expression. The combination treatment led to the most potent suppressive effect in Btk, p-Akt, mTOR and Nestin expression. **D.** The GBM sphere-generating ability was significantly affected by different treatments. The most effective sphere-inhibitory effect was by the combined treatment followed by Ib alone and TMZ. (10x magnification, scale bar = 100μm). The lower panels depict the percentage survival of GBM spheres under different treatment conditions. **E.** Western blots analysis of total cell lysates collected from GBM spheres after different treatments. Similarly, Btk, p-Akt, p-mTOR, Nestin and Vimentin expression level was suppressed by Ib treatment, while TMZ slightly increased p-Akt expression. The combination of Ib and TMZ (combo) exerted the most inhibitory effect. Treatment conditions: U87MG (Ib, 10 μM; TMZ 300 μM), DBTRG-05MG (Ib,30 μM, TMZ 500 μM).

Ibrutinib (Ib) is a FDA-approved Btk inhibitor for treating chronic lymphocytic leukemia [7]. Here, we intended to examine whether Ib treatment via a pharmacological means of down-regulating Btk, in GBM cells would yield similar anti-GBM activities as observed in Btk-silenced cells. As expected, Ib treatment suppressed key tumorigenic phenotypes. First, colony-forming ability in both U87MG (at 0.5μM) and DBTRG-05MG (at 10μM) cells was significantly reduced (Figure 4A). Second, the migratory ability of both cell lines was significantly reduced by ibrutinib treatment, approximately by 70% in U87MG (at 0.25μM) while by 30% DBTRG-05MG (at 4μM of ibrutinib) cells (Fig.4B).

Next, we demonstrated that by combining both ibrutinib and temozolomide, Btk expression and Akt/mTOR oncogenic pathways could be more pronouncedly suppressed (Figure 4C). Subsequently, the ability to generate GBM spheres in both cell lines was pronouncedly suppressed by the Ib treatment (Figure 4D), evident by the inability of both cell lines to form cell aggregates. Notably, the combination of relative low concentrations (less than their respective IC_50_ values in GBM sphere forming experiments) of Ib and TMZ appeared to supress the GBM sphere-forming ability in a greater extent than that of in single drug treatment (Figure 4E). The western blot analyses provided supports that the combined Ib and TMZ treatment yielded the most significant suppression in Btk, Akt/mTOR oncogenic pathway and EMT markers vimentin and nestin (also a neuro-stem cell marker).

### Suppression of GBM tumorigenesis via Btk downregulation in vivo

**Figure 5:**
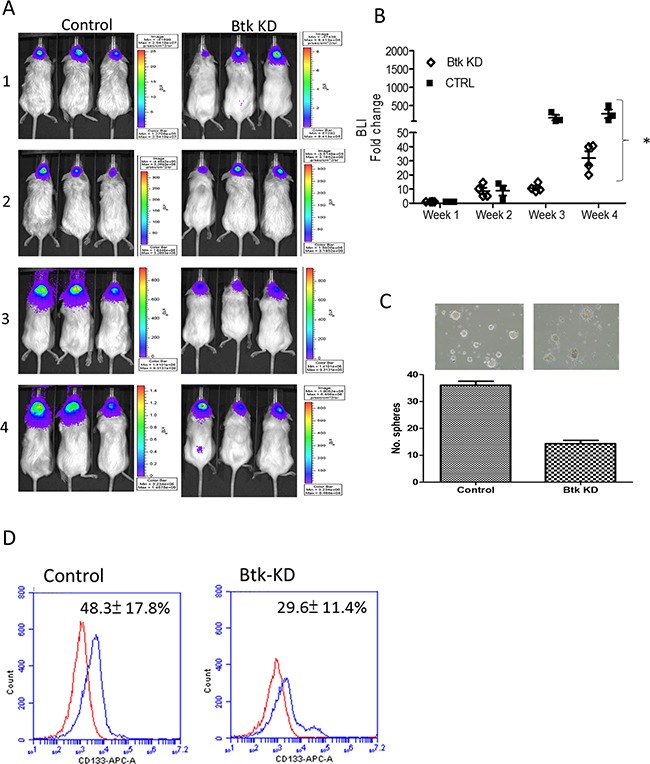
Btk-silencing led to decreased GBM tumorigenesis in vivo **A.** Representative bioluminescence imaging of wild-type Btk U87MG and Btk-silenced U87MG orthotopic xenograft mouse models. Btk-knockdown (KD) group clearly showed a lower bioluminescent signal over time indicating the delayed tumorigenesis. **B.** Semi-quantitative analysis of tumor growth represented by the fold change in bioluminescence intensity (BLI) over time. By week four post tumor implantation, it was clear that the control (CTRL) mice exhibited a significantly higher fold change in BLI as compared to those Btk-KD counterparts. **C.** Tumor biopsies were collected from both groups and Btk-KD samples demonstrated a significantly lower ability to generate GBM spheres as compared to those of CTRL counterparts. **D.** Comparative flow cytometry analysis showed that Btk-KD sample contained a significantly lower percentage of CD133+ cells as compared to its wild-type counterpart.

To provide preclinical support for targeting Btk as a mitigating strategy in glioma, GBM xenograft models were used. First, an orthotopic mouse brain tumor model was established by injecting firefly luciferase-expressing U87MG and Btk-silenced U87MG cells respectively. In vivo bioluminescence was used to monitor tumorigenesis over time (Figure 5A). Btk-silenced U87MG animals showed a significantly delayed tumorigenesis as reflected by the fold change in bioluminescence intensity (Figure 5B). Tumor biopsies from both groups were collected and cultured under serum deprived conditions to examine the GBM sphere-forming ability. Notably, Btk-silenced tumor sample showed a significantly decreased sphere-forming ability (Figure 5C).

### Ibrutinib treatment suppressed GBM tumorigenesis in vivo

**Figure 6:**
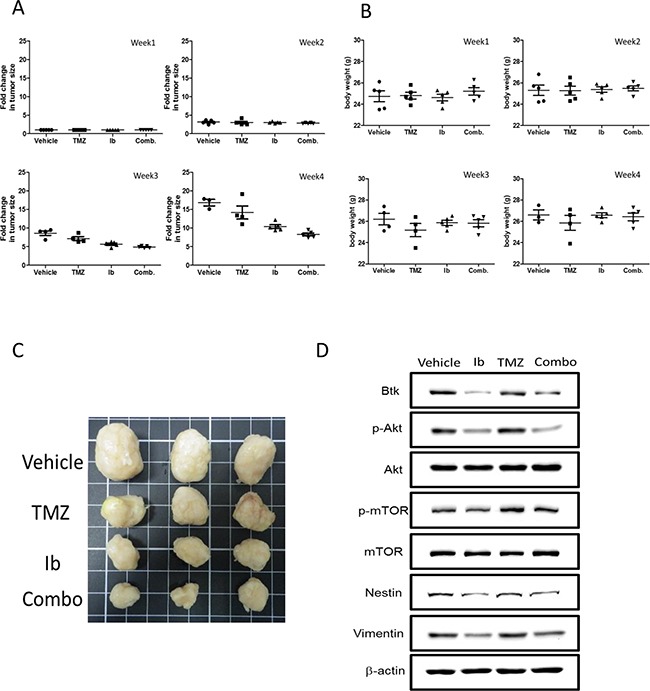
Evaluation of ibrutinib and temozolomide treatments using patient-derived xenograft mouse model **A.** Patient-derived GBM sample was injected subcutaneously into the right flank of NOD/SCID mice for evaluating the anti-GBM effect of ibrutinib (Ib) and temozolomide (TMZ). The fold change in tumor size was plotted against time. The tumor suppressive effect was found the most pronounced in the Ib+TMZ group followed by Ib alone, TMZ alone and vehicle control. The insert represents the photographs of tumor biopsies from different groups. **B.** The body weight of the mice was measured and tracked over time, demonstrating the treatments did not exert apparent toxicity in all groups. **C.** The photographic representation of tumor biopsies. **D.** Western blot analysis of the total lysates obtained from the tumor biopsies demonstrated the expression of Btk, members of mTOR signaling and stem marker, Nestin was suppressed by both Ib treatment alone and TMZ/Ib combination.

Next, we evaluated the potential usage of ibrutinib and in combination with TMZ in a patient-derived xenograft (PDX) mouse model. A GBM patient sample was collected and expanded in culture and subcutaneously injected into the right flank of NOD/SCID mice. Four groups of mice were established, the vehicle control, temozolomide (TMZ) only, ibrutinib (Ib) only and combination. The anti-tumorigenic effect was found to be in the following order, combination, Ib only, TMZ only and vehicle control (Figure 6A). Ib alone and Ib/TMZ combination treatment appeared to exert no apparent cytotoxic (no significant weight loss, Figure 6B).

### Clinical association of Btk to GBM malignancy

The role of Btk and the potential as a biomarker in GBM tumorigenesis was determined using tissue microarrays (TMAs). First, a TMA composing normal brain and glioma tissues were stained with anti-Btk antibody for evaluation. We found that all 4 cases of grade IV GBM exhibited strong Btk staining while none of the normal brain tissue did (Table 1). It is noteworthy that T-cell lymphoma on the TMA also showed strong Btk reactivity, serving as a positive control. More importantly, in a TMA containing 33 cases of glioma samples (duplicates, N=66), 2 anaplastic astrocytoma (duplicates, N=4), 11/59 GBM spots showed moderate Btk staining and 35/59 showed strong staining, suggesting 77.9% of the GBM samples stained for Btk (Table 2).

**Table 1:** Btk expression in brain tumor tissue microarray

Sample information		No. of cases Sample size	Immunohistochemical staining		
			None/weak	Moderate	Strong
Gender					
	Male	12	5	1	6
	Female	12	7	4	1
Age	>60	4	1	3	0
	<=60	20	11	2	7
Grade	II	4	4	0	0
	III	2	1	1	0
	IV	4	0	0	4
Tumor type	Normal	4	4	0	0
	Benign	4	3	1	0
	Malignant	16	5	3	8
Pathology	Oligo-astrocytoma	2	2	0	0
	Astrocytoma	2	2	0	0
	Glioblastoma	4	0	0	4
	Glioma sarcomatosum	2	0	2	0
	Malignant ependymoma	2	1	1	0
	T-cell lymphoma	2	0	0	2
Meningioma					
	Transitional	2	2	0	0
	Malignant	2	0	1	1
	Epithelial	2	1	1	0
Normal brain tissue		4	4	0	0

A small cohort of brain tumor tissue microarray containing brain tumor and normal brain tissue (N=24) was stained with anti-Btk antibody. We found that 8/16 malignant brain tumor exhibited strong Btk staining while very weak or no staining was found in the normal brain tissue. Btk expression level was also correlated to the stage of disease where all grade IV GBM stained strong with anti-Btk antibody while 1/4 grade III with moderated Btk signal.

**Table 2:** Btk expression analysis in GBM tissue microarray

Samples	Description	No. of cases	Immunohistochemical staining		
			None/Weak	Moderate	Strong
Gender					
	Male	40	11	9	20
	Female	39	7	7	25
Age					
	>60	6	3	1	2
	<=60	73	15	15	43
Tissue type					
	Normal	9	6	3	0
	Malignant	70	16	15	39
Pathology	Glioblastoma	59	13	11	35
	Glioblastoma (Cataplasia)	3	1	2	0
	Glioblastoma (Necrosis)	1	0	0	1
	Anaplastic astrocytoma (Grade III)	4	1	1	2

This GBM tissue microarray contains 33 cases of GBM, 2 anaplastic astrocytoma and 5 normal brain tissue, duplicate cores per case. We found that 35/59 GBM samples with strong Btk signal and 2/4 grade V

## DISCUSSION

The cancer stem-cell theory entails that a subpopulation of transformed stem cells, or progenitors with acquired self-renewal properties, represent the source of tumor-initiation epithelial-to-mesenchymal transition and repopulation post treatment. Several seminal studies demonstrated CD133+ glioma cells exhibited enhanced ability to evade treatment and increased EMT incidence leading to distant metastasis, both contributing to the high mortality of patients with high grade glioblastoma [11, 14]. Thus, targeting GSCs has emerged as a prime target for anti-GBM drug development and the identification of markers associated with the generation and/or maintenance as an imminent task.

In this study, we have shown that Bkt have many attributes as a therapeutic/prognostic marker for GBM. First, a significantly higher Btk expression is detected in clinical glioma samples from different public databases where Btk expression is generally low in most tissues except for the hematopoietic lineages and organs such as lung and spleen. High Btk expression is well established in B-cell malignancies such as chronic lymphocytic leukemia (CLL) and mantle cell lymphoma (MCL); inhibition of Btk-signaling pharmacologically by FDA-approved Btk inhibitor, ibrutinib, has been shown to be effective in managing these diseases [7, 15]. According to our database analyses, Btk mRNA level is several fold higher in astrocytoma, glioblastoma and secondary glioblastoma clinical samples as compared to the normal astrocytes. More importantly, in our TMA analysis not only demonstrated that strong Btk staining in different brain tumor samples but also correlated Btk staining to a more malignant GBM phenotype (mostly in stage IV). In support to our view, BMX a close family member of Btk was recently found to be elevated in GBM samples and enriched in GSCs [8]. These findings support our hypothesis where non-receptor tyrosine kinase Btk/BMX pathway plays a key role in GBM tumorigenesis. More importantly, our analysis indicates that normal brain tissues express very low level of Btk, making Btk an ideal target and specific biomarker for glioma

Next, we demonstrated the functional roles of Btk in GBM cell lines using gene-silencing technique. Btk-silenced GBM cells exhibited a significantly decreased malignant phenotypes where colony formation, migration and TMZ resistance were pronouncedly suppressed as compared to their parental counterparts. More importantly, Btk-silenced GBM cells appeared to be significantly less efficient in forming GBM spheres. Btk-silencing was associated with the down-regulation of major stemness and oncogenic markers. For instance, the most studied stemness marker for a spectrum of cancer stem cells, particularly in GSCs, CD133 [4, 11], was significantly suppressed in the wake of Btk-silencing. Nestin, an important neural stem cell marker and EMT indicator, has been shown to be highly expressed in GBM patients with increased stemness and associated with a poor prognosis [16, 17]; Nestin was also significantly suppressed in Btk-silenced GBM cells. The down-regulation of Akt/mTOR pathway mediated by Btk-silencing and ibrutinib-treatment marks one of the most important observations in this study. It has been shown that the activation of Akt signaling leads to increased proliferation and survival of GBM cells [18, 19], and Akt/mTOR signaling has been targeted for glioma therapeutics [20]. Together, our data strongly suggest that Btk plays a pivotal role in GBM tumorigenesis and GSC generation/maintenance. Previous report on BMX, another tec-family non-receptor tyrosine kinase, also found BMX to be pivotal for GSC generation and maintenance but through STAT3 signaling [8]. In addition, we also observed that suppression of Btk either by gene silencing or pharmacological inhibition by ibrutinib treatment led to a pronounced c-Myc suppression (data not shown). This finding lends additional support towards repurposing ibrutinib for GBM treatment. Since c-Myc up-regulation is tightly associated with deranged metabolism in many cancer types including GBM [20, 21], targeting Btk may provide another venue for restoring normal metabolic function in GBM cells and increase their response towards chemotherapeutic agents. Currently, this topic is under investigation in our laboratory.

Repurposing drug represents one of the most economic and rapid way for developing much required cancer fighting interventions [22, 23]. Here, we provided preclinical evidence that FDA-approved ibrutinib exerted several anti-GBM activities including suppression in colony formation, migration, and re-sensitization of TMZ. More importantly, ibrutinib treatment render GBM cells incapable of forming GBM spheres (cancer stem-like cells), all of which are new indications from ibrutinib's original role as a B cell malignancy antagonist. The positive results from our patient-derived xenograft (PDX) GBM mouse study provides important preclinical evidence for the use of ibrutinib to treat GBM patients. More importantly, ibrutinib has been shown to effectively cross the blood-brain-barrier [24], adding more support for future clinical use of ibrutinib for treating GBM patients. We realize the limitation of the small number of our clinical sample and more clinical samples are being collected for future studies.

A recent study demonstrated that TMZ treatment is associated with an AKT-induced NF-kB activation leading to decreased sensitivity and protection against TMZ [25]. This observation was also made in our study (data not shown). Our finding where ibrutinib could suppress Akt/mTOR signalling provides the support for using ibrutinib in combination with TMZ to prevent TMZ-associated induction of Akt/mTOR signaling. This may provide partial explanation for the effectiveness of ibrutinib and temozolomide combination in inhibiting the generation of GBM spheres.

Collectively, we have provided a novel association between Btk and GBM malignancy first by bioinformatics approach followed by in vitro and in vivo validation. Notably, clinical tissue arrays revealed that Btk expression was associated with the later stage of the disease. The role of Btk in GSC generation and ability to resist temozolomide treatment via the increased expression of several key stemness/onocogenic molecules including CD133, Nestin, and phosphorylated Akt was also demonstrated. Perceptively, we provide preclinical evidence to support the usage of ibrutinib with temozolomide for the treatment of GBM in mouse xenograft models. This combination may provide improved therapeutic efficacy for GBM patients in clinical settings.

## MATERIALS AND METHODS

### Cell culture

Human malignant glioma cell lines U87MG (ATCC^®^ HTB-14™) and DBTRG-05MG (ATCC^®^ CRL-2020™) were purchased from American Type Culture Collection (ATCC) for this study. U87MG is categorized as glioblastoma; astrocytoma; classified as grade IV by ATCC. The DBTRG-O5MG (Denver Brain Tumor Research Group 05) cell line was established from the tumor tissue of a GBM patient who had been treated with local brain irradiation and multidrug chemotherapy. U87MG were grown in complete Dulbecco's modified Eagle medium (DMEM) (GIBCO) and Roswell Park Memorial Institute (RPMI) (Sigma-Aldrich), supplemented with 10 % fetal bovine serum (GIBCO) in an incubator with 5 % CO2, respectively. Ibrutinib (PCI-32765) and temozolomide stocks (SelleckChem, Taiwan) were dissolved in DMSO. The final concentration of DMSO in the culture medium should be adjusted to be below 0.01 % and not affect the cell viability and the expression of the proteins.

### Isolation of CD133+ GSCs using FACS and generation of GBM spheres

Flow cytometry was used to profile GBM cells using the BD Accuri™ C6 personal flow cytometer. CD133/1 (AC133) antibodies conjugated to APC (Miltenyi Biotec, Auburn, CA, USA) was used to identify CD133+ GBM cells. Subsequently, GBM cells were labeled and sorted using magnetic microbeads (Isotype antibodies served as control (all from Coulter-Immunotech Co., Miami, FL, USA). Live cells were sorted and then analyzed by using FACSAria Cell Sorter unit (Becton Dickinson), after adding propidium iodide (PI). Cells were gated with the parameters of low side scatter, low-to-moderate forward scatter, and low PI. In routine, at least 10,000 events were analyzed. Approximately >95% of isolated cells were viable. GBM spheres were generated according to established protocol [26] with slight modifications. Parental cells (10^5^ cells) were seeded in Corning ultra-low attachment plates (Sigma, USA) with a serum-free stem cell medium (Nutristem, Biological Industries, Israel) at 37°C in a 5% CO2 incubator for at least 2-7 days when spheres were visible (>50μM in diameter) under a microscope. At least three passages were performed to obtain GSCs for analyses.

### Cell viability assay

Cellular viability was tested using SRB assay as established previously [27]. Briefly, GBM cells and/or spheres were seeded in 96-well plates (3.5 × 10^5^ cells/well) and treated with drugs of interest (temozolomide, ibrutinib) alone or in combination at indicated concentrations and times. Upon harvest, the relative cell number was calculated using a SRB reagent according to the manufacturer's protocol (Sigma, USA). The viability of non-attached cells in GBM cells and/or spheres were quantified using Alamar blue staining (Life Technologies, USA).

### SDS-PAGE and western blotting

Total protein lysates of GBM cells and spheres were obtained using a protein extraction Kit (Panomics, Fremont, CA, USA). Protein samples (20 μg/sample) were separated using SDS-PAGE and transferred onto a PVDF membrane via BioRad Mini Protean electrotransfer system. The transferred Blots were subsequently blocked with 5% skim milk in PBST (PBS plus Tween) and incubated with primary antibodies, Btk, mTOR, Akt (and their phosphorylated forms denoted as p-mTOR and p-Atk), Santa Cruz Biotechnology Inc., Taiwan) overnight at 4°C. Respective peroxidase-conjugated secondary antibodies were added and protein signals were developed with the use of the ECL detection kit. The developed images were obtained and analyzed using UVP BioDoc-It system (Upland, CA, USA).

### Migration assay

The migratory ability of CD133+ and parental GBM cells was measured using Transwell migration assay (ThermoFisher, Taipei, Taiwan). Briefly, cells were trypsinized, washed with PBS buffer, and re-suspended in a serum-free DMEM medium (cells/200 uL) with or without drugs (Ib, TMZ). The cells were then seeded into the upper chamber with 8 mm pore polycarbonate filters. A serum-containing DMEM medium (500 μL) was added to the lower chambers. After 24 h of incubation, medium was discarded, cells on filter membrane were further fixed with 3.7 % formaldehyde solution and then stained with crystal violet. The remaining cells on the upper side of the insert were removed. The migrated cells were examined and counted. And the migratory capacity was determined as the total number of cells on the lower side of membrane.

### Xenograft GBM model and drug treatment

Two GBM xenograft models were established in this study. First, U87MG parental and Btk-silenced U87MG cells (1x10^5^ cells/injection) were orthotopically injected into NOD/SCID mice using previously established protocol [28]. Tumorigenesis was monitored using bioluminescence (IVIS 200 system, Caliper) on a weekly basis. The tumor burden was measured using Living Imaging software. The change in tumor burden was indicated using fold change in bioluminescence intensity over time. Second, in the subcutaneous tumor model, a patient-derived GBM sample was used. The sample was obtained under the strict adherence to the regulations stated in the TMU-IRB regulations (IRB number: MOHW105-TDU-B-212-134001). The sample was first expanded in vitro and subcutaneously into the right flank of NOD/SCID mice allowed to grow until palpable and treatment was initiated. The dosage of temozolomide (TMZ, 42mg/kg, oral gavage, 5 times/week) used in this study was based on previously established protocol [29] while ibrutinib was given intraperitoneally, 6mg/kg, 5 times/week. Tumor volume was measured weekly using a standard caliper. The change in tumor burden was expressed in fold change in mm^3^ as compared to its starting volume. Mice were humanely sacrificed at the end of the experiment.

### Tissue microarray analysis

We further strengthened the association between Btk expression and GBM tumorigenesis using commercially available tissue microarrays. The first array is a brain tumor tissue array, containing 10 cases of brain tumor and 2 normal brain tissues, duplicate cores per case (category # T175 A049, US Biomax, Inc. Rockville, MD). The second array is a glioblastoma multiforme tissue array with several normal brain tissues as control. The pathology grade was included. A total of 40 cases in 80 cores was spotted on the array slide. (category # GL806c, US Biomax, Inc. Rockville, MD) Both arrays were stained with anti-Btk antibody (1:1000) using standard immunohistochemical protocol established previously [30].

## SUPPLEMENTARY FIGURES


