# Supplementary material for: Preclinical investigation of ibrutinib, a Bruton's kinase tyrosine (Btk) inhibitor, in suppressing glioma tumorigenesis and stem cell phenotypes
Source: Oncotarget. 2016 Aug 24;7(43):69961–75. doi: 10.18632/oncotarget.11572 (PMC5342527; doi:10.18632/oncotarget.11572)
Supplement: Supplementary file 1 [file oncotarget-07-69961-s001.pdf]

# Preclinical investigation of ibrutinib, a Bruton's kinase tyrosine (Btk) inhibitor, in suppressing glioma tumorigenesis and stem cell phenotypes

## SUPPLEMENTARY FIGURES

A

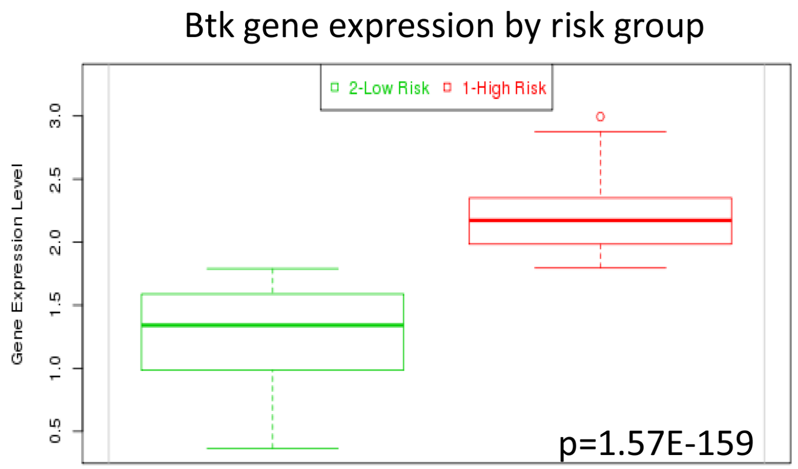


B

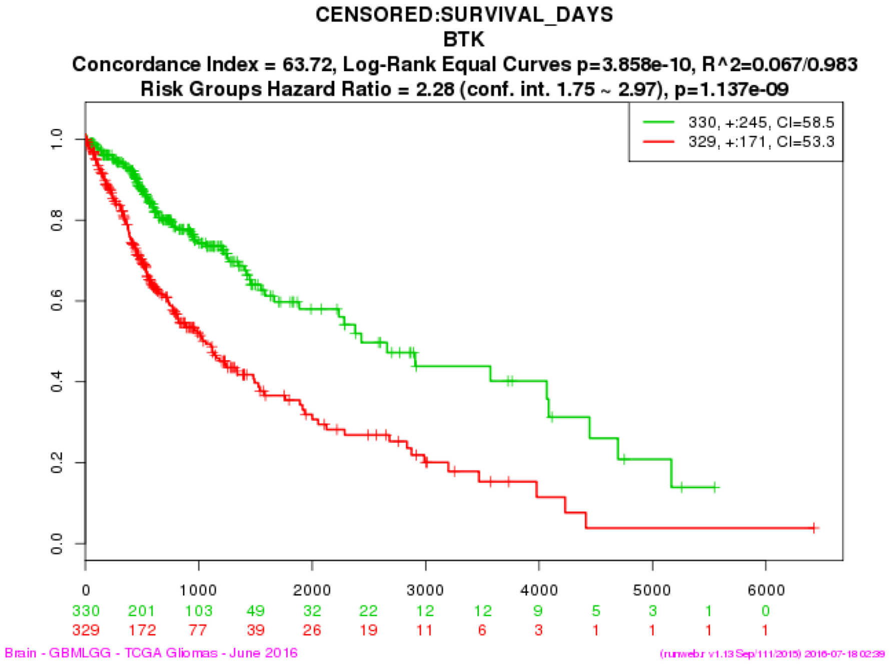


**Supplementary Figure S1: Poor prognosis is associated with high Btk expression. A.** Using TCGA glioma dataset (GBMLGG, N=660), we found that a significant higher Btk expression was found in glioma patients with high risk (red). **B.** A shorter patient survival was also found significantly correlated with Btk expression in the same dataset.

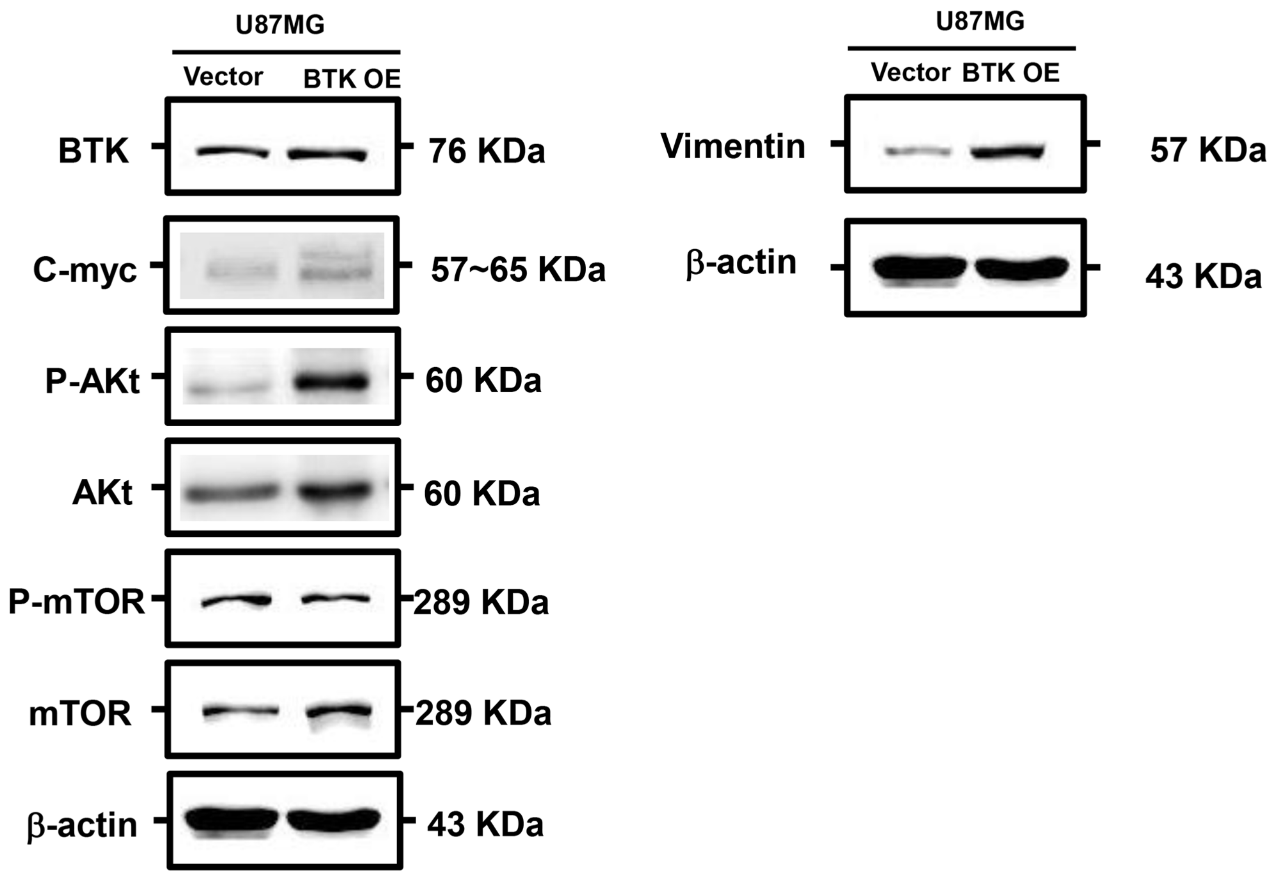


**Supplementary Figure S2: Ectopic overexpression of Btk in U87MG cells led to increased c-Myc, Akt/mTOR signaling and vimentin expression.**
